# Supplementary material for: Conflicting effects of recombination on the evolvability and robustness in neutrally evolving populations
Source: PLoS Comput Biol. 2022 Nov 21;18(11):e1010710. doi: 10.1371/journal.pcbi.1010710 (PMC9721492; doi:10.1371/journal.pcbi.1010710)
Supplement: S1 Appendix — (PDF) [file pcbi.1010710.s001.pdf]

Conflicting effects of recombination on the evolvability and robustness in neutrally evolving populations

Alexander Klug[1,2] and Joachim Krug[1]

[1]Institute for Biological Physics, University of Cologne, Cologne, Germany

[2]Current Address: Institute of Integrative Biology, ETH Zurich, 8092 Zurich, Switzerland

SUPPLEMENTARY APPENDIX

In the following, analytical expressions for the mean Hamming distance $\overline{d}_{pw}$ are derived for all three recombination models illustrated in Fig. 1. Throughout this appendix these models are abbreviated as *cr* (concurrent recombination), *ssr* (simple successive recombination) and *srmp* (successive recombination with mating pairs), respectively. The derivation is based on the approach of [1], which is generalized to take into account recombination. It is assumed that there are no lethal genotypes ($p = 1$). The results apply to the *fsm* and the *ism* and show that the recombination rate influences $\overline{d}_{pw}$ only in the *ssr* model. The r-dependence decreases with N in the *fsm* but remains independent of N in the *ism*.

To start, in the selection step each individual α picks a parent α' at random from the previous generation. During the mutation step, a mutation occurs at each locus i with probability μ, changing its state from -1 to 1 or vice versa,

$$\sigma_i^\alpha(t+1) = \begin{cases} \sigma_i^{\alpha'}(t), & \text{with prob. } 1-\mu \\ -\sigma_i^{\alpha'}(t), & \text{with prob. } \mu. \end{cases} \tag{A1}$$

This can also be written as

$$\sigma_i^\alpha(t+1) = \epsilon_i^\alpha(t)\sigma_i^{\alpha'}(t) \tag{A2}$$

with

$$\epsilon_i^\alpha(t) = \begin{cases} +1, & \text{with prob. } 1-\mu \\ -1, & \text{with prob. } \mu. \end{cases} \tag{A3}$$

During the recombination step, individuals recombine at rate r. Altogether this leads to

$$\begin{aligned} \sigma_i^\alpha(t+1) = &\epsilon_i^\alpha(t)\Big[\kappa^\alpha(t)\left(\xi_i^\alpha(t)\sigma_i^{\alpha'}(t) + (1-\xi_i^\alpha(t))\sigma_i^{\alpha''}(t)\right) \\ &+(1-\kappa^\alpha(t))\sigma_i^{\alpha'''}(t+1)\Big] \end{aligned} \tag{A4}$$

where α', α'' are the parental genotypes in case of recombination and α''' is the parent in case of no recombination. The random variable κ^α determines whether a recombination event occurs and is given by

$$\kappa^\alpha(t) = \begin{cases} 1 & \text{with prob. } r, \\ 0 & \text{with prob. } 1-r. \end{cases} \tag{A5}$$

The random variable ξ_i^α determines from which parent the allele is taken in case of a recombination event:

$$\xi_i^\alpha(t) = \begin{cases} 1 & \text{with prob. } 1/2, \\ 0 & \text{with prob. } 1/2. \end{cases} \tag{A6}$$

Next the relatedness Q of the population is computed, which is defined by

$$Q = \binom{N}{2}^{-1} \sum_{(\alpha,\beta)} \frac{1}{L}\sum_{i=1}^{L} \sigma_i^\alpha\sigma_i^\beta \tag{A7}$$

where the sum runs over all different pairs of individuals (α,β). We are interested in the average relatedness $\overline{Q}$ in the stationary state, and therefore make use of Eq. A4 by evaluating

$$\begin{aligned}
&\overline{\sigma_i^\alpha(t+1)\sigma_i^\beta(t+1)} \\
&= \underbrace{\epsilon_i^\alpha\epsilon_i^\beta}_{(1-2\mu)^2}\Big[\underbrace{\kappa^\alpha\kappa^\beta}_{r^2}\underbrace{\overline{\left(\xi_i^\alpha\sigma_i^{\alpha'}+(1-\xi_i^\alpha)\sigma_i^{\alpha''}\right)\left(\xi_i^\beta\sigma_i^{\beta'}+(1-\xi_i^\beta)\sigma_i^{\beta''}\right)}}_{(*)} \\
&\quad \overline{+\underbrace{(1-\kappa^\alpha)(1-\kappa^\beta)}_{(1-r)^2}\sigma_i^{\alpha'''}\sigma_i^{\beta'''}} \\
&\quad \overline{+\underbrace{\kappa^\alpha(1-\kappa^\beta)}_{r(1-r)}\underbrace{\left(\xi_i^\alpha\sigma_i^{\alpha'}+(1-\xi_i^\alpha)\sigma_i^{\alpha''}\right)\sigma_i^{\beta'''}}_{\overline{\sigma_i^{\alpha'}\sigma_i^{\beta'''}}}} \\
&\quad \overline{+\underbrace{(1-\kappa^\alpha)\kappa^\beta}_{r(1-r)}\underbrace{\sigma_i^{\alpha'''}\left(\xi_i^\beta\sigma_i^{\beta'}+(1-\xi_i^\beta)\sigma_i^{\beta''}\right)}_{\overline{\sigma_i^{\beta'}\sigma_i^{\alpha'''}}}}\Big].
\end{aligned} \tag{A8}$$

To simplify notation, the t-dependence is suppressed on the right hand side. Equation A8 holds for all three models, but differences emerge in the probability that the individuals α, β share a parent as well as in the term marked by $(*)$.

The probability that two non-recombining individuals have the same parent $\alpha''' = \beta'''$ through selection is given by $1/N$ in all models. This leads to

$$\overline{\sigma_i^{\beta'''}(t)\sigma_i^{\alpha'''}(t)} = \frac{1}{N} + \left(1-\frac{1}{N}\right)\overline{Q}(t). \tag{A9}$$

With *cr* and *srwm*, a recombining and non-recombining individual share parents $\alpha' = \beta'''$ with probability $1/N$, leading again to the right hand side of Eq. A9. However, for *ssr*, the probability that they share a common parent $\alpha' = \beta'''$ is $1/N + (1-1/N)1/N \approx 2/N$, as this can occur either during the recombination or selection step. This yields

$$\overline{\sigma_i^{\beta'''}(t)\sigma_i^{\alpha'}(t)} = \overline{\sigma_i^{\beta'}(t)\sigma_i^{\alpha'''}(t)} \approx \frac{2}{N} + \left(1-\frac{2}{N}\right)\overline{Q}(t). \tag{A10}$$

We next turn to the evaluation of the term in Eq. A8 marked by $(*)$. In the *cr* and *ssr* model the random variables ξ_i are not correlated between individuals. Therefore in both models $(*)$ simplifies to

$$(*) = \overline{\sigma_i^{\alpha'}(t)\sigma_i^{\beta'}(t)}. \tag{A11}$$

For *cr* this leads to the right hand side of Eq. A9, while for *ssr* we get Eq. A10 using the same argument as before. Similar to *ssr*, for *srwm*, recombining individuals have an increased chance of sharing a parent $\alpha' = \beta'$ since they either can belong to the same mating pair with probability $2/(rN)$ or share a parent during selection with probability $1/N$. However, this is exactly balanced by the constraint that mating pairs are complementary in their recombined material, which is reflected in a correlation of the random variables ξ_i. Simply put, the increased chance that two individuals share the same parent is offset by the constraint that they always inherit the allele of the respective other parental genotype. Therefore $(*)$ again leads to Eq. A9 for *srwm*.

Summarizing, we have

$$\begin{aligned}
\text{suc. rec. pairs:}\quad \overline{Q(t+1)} &= (1-2\mu)^2\left[\frac{1}{N}+(1-\frac{1}{N})\overline{Q}(t)\right]. \\
\text{conc. rec.:}\quad \overline{Q(t+1)} &= (1-2\mu)^2\left[\frac{1}{N}+(1-\frac{1}{N})\overline{Q}(t)\right]. \\
\text{simp. suc. rec.:}\quad \overline{Q(t+1)} &\approx (1-2\mu)^2\Big[r^2(\frac{2}{N}+(1-\frac{2}{N})\overline{Q}(t)) \\
&\quad +(1-r)^2(\frac{1}{N}+(1-\frac{1}{N})\overline{Q}(t)) \\
&\quad +2r(1-r)(\frac{2}{N}+(1-\frac{2}{N})\overline{Q}(t))\Big].
\end{aligned} \tag{A12}$$

Note that the r dependence has vanished with *cr* & *srmp*. Next we compute the stationary relatedness by setting $\overline{Q(t+1)} = \overline{Q(t)}$. For *cr* & *srmp* this yields

$$\overline{Q} = \frac{(1-2\mu)^2}{4(1-\mu)\mu(N-1)+1}, \tag{A13}$$

while for *ssr* we get

$$\overline{Q} = \frac{(1-2\mu)^2[1+r(2-r)]}{4(1-\mu)\mu(N-1)-(1-2\mu)^2r^2+2(1-2\mu)^2r+1}. \tag{A14}$$

The relatedness is connected to the mean Hamming distance through

$$\overline{d}_{pw} = \frac{L(1-\overline{Q})}{2}, \tag{A15}$$

which leads in the cases of *cr* & *srmp* to

$$\overline{d}_{pw} = \frac{2(1-\mu)\mu LN}{4(1-\mu)\mu(N-1)+1} \tag{A16}$$

and in the case of *ssr* to

$$\overline{d}_{pw} = \frac{2(1-\mu)\mu LN}{4(1-\mu)\mu(N-1)-(1-2\mu)^2r^2+2(1-2\mu)^2r+1}. \tag{A17}$$

In Figures S10 and A1 we compare the expressions in Eqs. A16 and A17 to numerical simulations and find excellent agreement.

In the deterministic limit $N \to \infty$ with finite L the mean Hamming distance is the same for all recombination models:

$$\overline{d}_{pw} = \frac{L}{2}. \tag{A18}$$

However, in the *ism* limit $(L \to \infty, \mu \to 0, L\mu = U)$ the result for *cr* & *srmp* leads to

$$\overline{d}_{pw} = 2NU = \theta \tag{A19}$$

while the result for *ssr* reads

$$\overline{d}_{pw} = \frac{\theta}{1+r(2-r)}. \tag{A20}$$

Hence in this limit the dependence on the recombination rate persists independent of the population size.

To obtain an analytical expression for $\overline{S}$ in the *ism* under *ssr* we adopt a relation between the mean Hamming distance and the number of segregating sites $\overline{S}$ that has been derived for the non-recombining case [2]. This leads us to

$$\overline{S} \approx \overline{d}_{pw} \sum_{i=1}^{N-1} \frac{1}{i} = \frac{\theta}{1+r(2-r)} \sum_{i=1}^{N-1} \frac{1}{i}. \tag{A21}$$

Figure A1 shows that, at least for the parameter regime of interest here, Eq. A21 provides an accurate approximation of the simulation results.

[1] M. Serva and L. Peliti, Journal of Physics A: Mathematical and General **24**, L705 (1991).
[2] J. Wakeley, *Coalescent theory: An introduction* (Roberts & Co. Publishers, 2009).

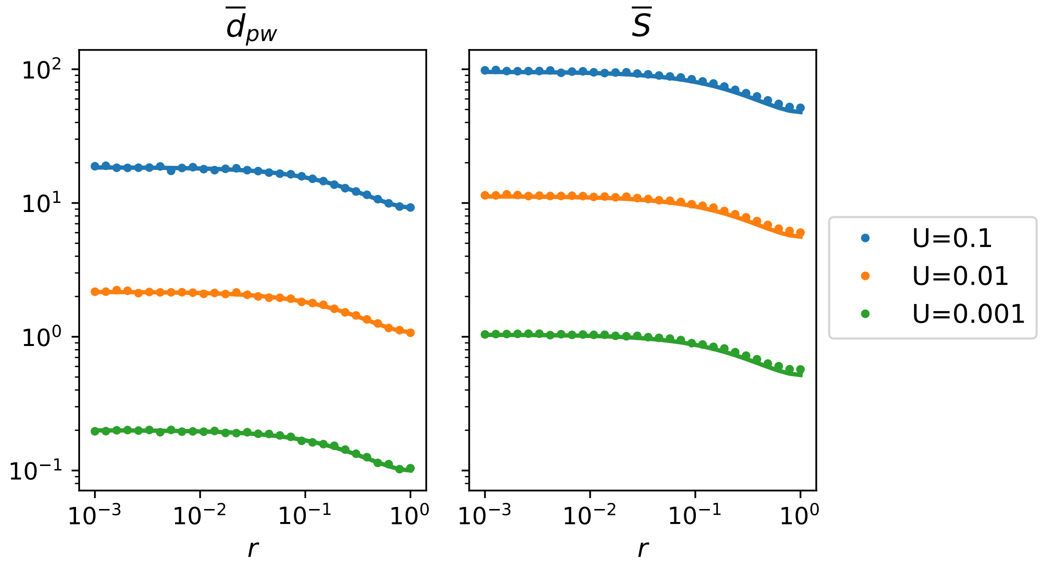

Fig A1. **Comparison of numerical results for $\overline{d}_{pw}$ and $\overline{S}$ to the analytical expressions for the simple successive recombination model.** Dots represent numerical results while lines represent Eqs. A17 and A21. Simulations were carried out using the *ism* with population size $N = 100$ and three different mutation rates. While for $\overline{d}_{pw}$ the fit is perfect, for $\overline{S}$ some deviations are discernible at large r.
