# Supplementary material for: Conflicting effects of recombination on the evolvability and robustness in neutrally evolving populations
Source: PLoS Comput Biol. 2022 Nov 21;18(11):e1010710. doi: 10.1371/journal.pcbi.1010710 (PMC9721492; doi:10.1371/journal.pcbi.1010710)
Supplement: S1 Fig — Genotypes are viable or lethal independently with probability p. The sequence length is L = 10. (PDF) [file pcbi.1010710.s002.pdf]

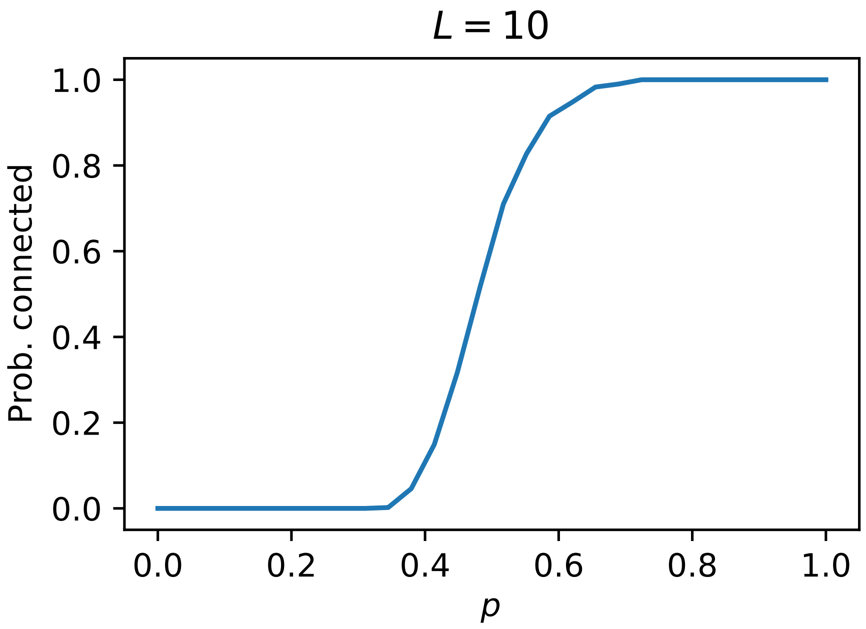

FIG. S1. **Probability that the network of viable genotypes is connected in a percolation landscape.** Genotypes are viable or lethal independently with probability p. The sequence length is $L = 10$.
