# Supplementary material for: Conflicting effects of recombination on the evolvability and robustness in neutrally evolving populations
Source: PLoS Comput Biol. 2022 Nov 21;18(11):e1010710. doi: 10.1371/journal.pcbi.1010710 (PMC9721492; doi:10.1371/journal.pcbi.1010710)
Supplement: S2 Fig — The right column with U = 0.1 is identical to that of Fig 9, and the left column illustrates the behavior at mutation rate U = 0.05. The results show that at lower mutation rates the contraction of the genotype cloud occurs already at larger fractions of viable genotypes (p = 0.7) and moreover shifts to smaller recombination rates. At the same time the magnitude of the effect of recombination becomes smaller for smaller U. At even lower mutation rates the population becomes monomorphic and recombination has no effect. (PDF) [file pcbi.1010710.s003.pdf]

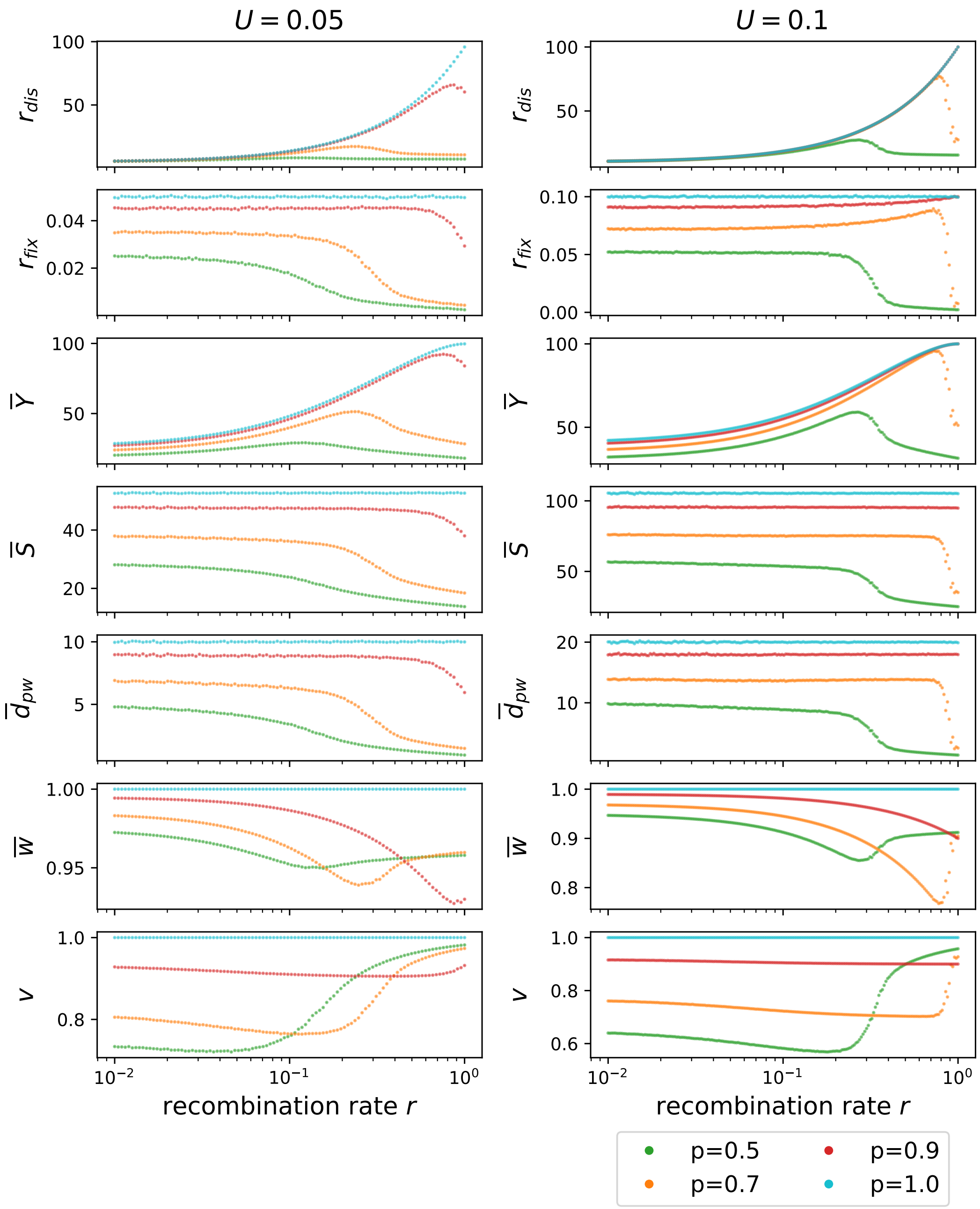

FIG. S2. **Supplementary information to Fig. 9.** The right column with $U = 0.1$ is identical to that of Fig. 9, and the left column illustrates the behavior at mutation rate $U = 0.05$. The results show that at lower mutation rates the contraction of the genotype cloud occurs already at larger fractions of viable genotypes ($p = 0.7$) and moreover shifts to smaller recombination rates. At the same time the magnitude of the effect of recombination becomes smaller for smaller U. At even lower mutation rates the population becomes monomorphic and recombination has no effect.
