# Supplementary material for: Conflicting effects of recombination on the evolvability and robustness in neutrally evolving populations
Source: PLoS Comput Biol. 2022 Nov 21;18(11):e1010710. doi: 10.1371/journal.pcbi.1010710 (PMC9721492; doi:10.1371/journal.pcbi.1010710)
Supplement: S3 Fig — The population size is N = 1000 (left panel) vs. N = 100 (right panel) with p = 0.5. The green line is drawn at U = 0.1 and the blue lines in both panels show Eq 11. The right panel is identical to that in Fig 2. (PDF) [file pcbi.1010710.s004.pdf]

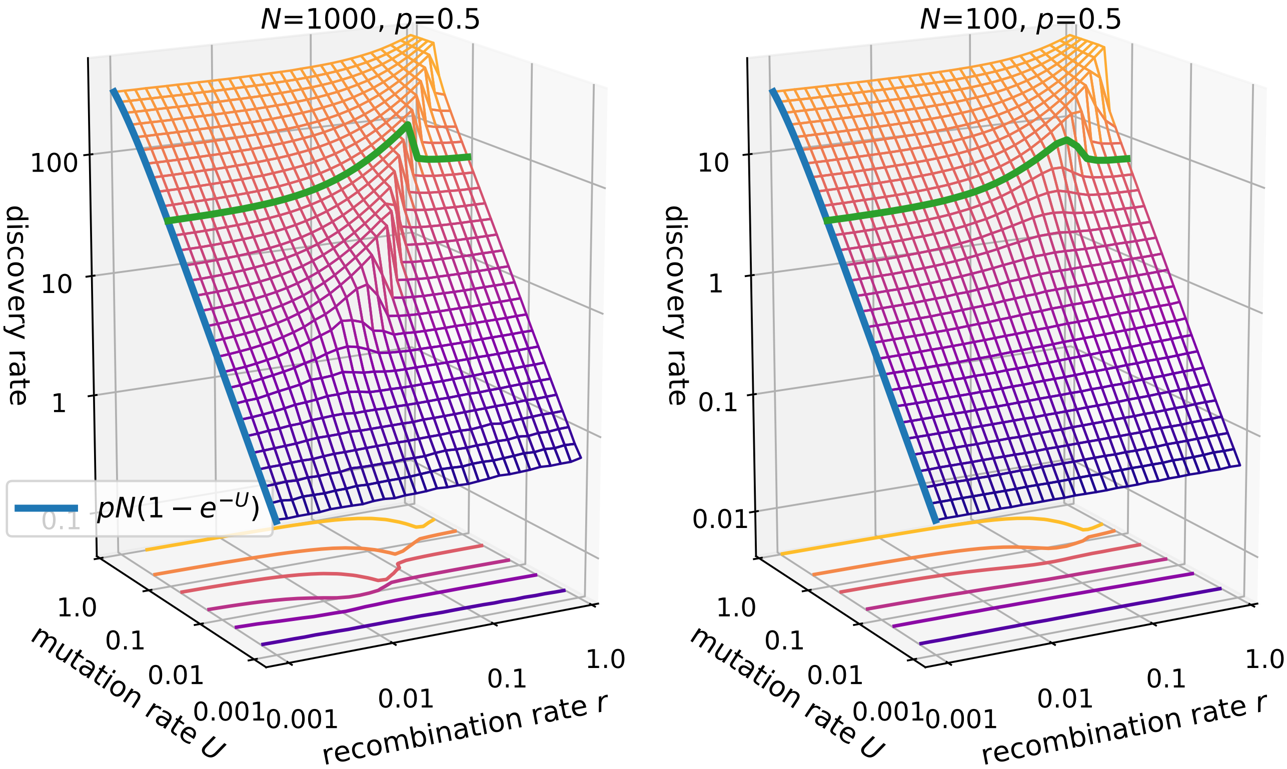

FIG. S3. **Discovery rate in the *ism* for two different population sizes.** The population size is $N = 1000$ (left panel) vs. $N = 100$ (right panel) with $p = 0.5$. The green line is drawn at $U = 0.1$ and the blue lines in both panels show Eq. 11. The right panel is identical to that in Fig. 2.
