# Supplementary material for: Conflicting effects of recombination on the evolvability and robustness in neutrally evolving populations
Source: PLoS Comput Biol. 2022 Nov 21;18(11):e1010710. doi: 10.1371/journal.pcbi.1010710 (PMC9721492; doi:10.1371/journal.pcbi.1010710)
Supplement: S4 Fig — The figure shows the results of simulations for U = 0.05 and p = 0.7 in which the fitness of the unfit genotypes is varied between w0 = 0 and w0 = 0.6, whereas high fitness genotypes have fitness w1 = 1. The contraction of the genotype cloud at large recombination rates is seen to persist up to w0 = 0.4 for the chosen parameters, showing that lethality of the deleterious mutations is not a necessary condition for the results presented in the main text. (PDF) [file pcbi.1010710.s005.pdf]

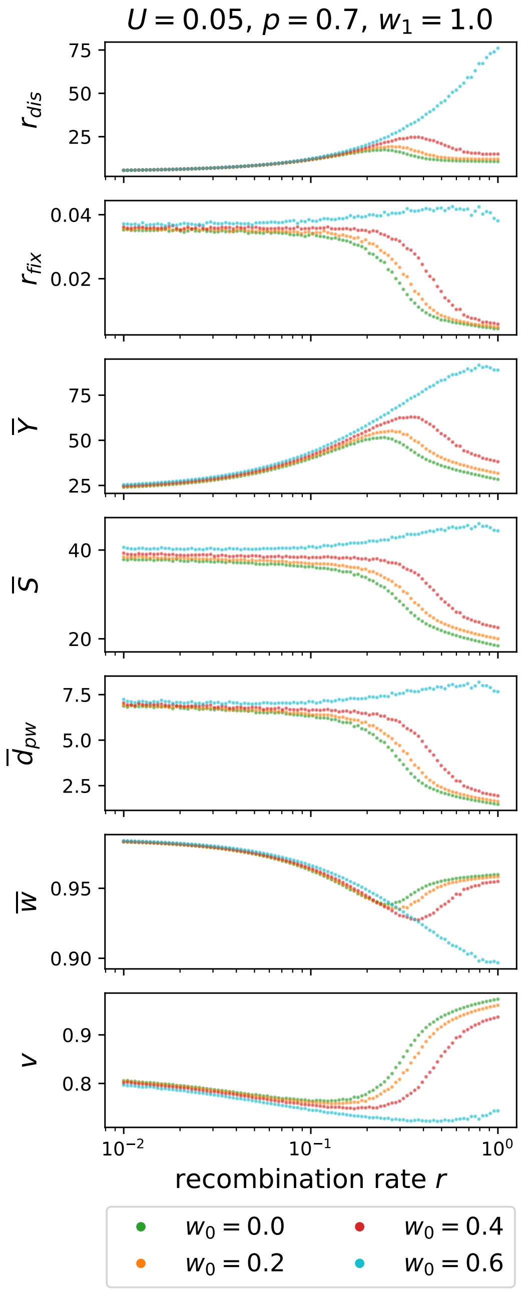

FIG. S4. **Varying fitness of unfit genotypes.** The figure shows the results of simulations for $U = 0.05$ and $p = 0.7$ in which the fitness of the unfit genotypes is varied between $w_0 = 0$ and $w_0 = 0.6$, whereas high fitness genotypes have fitness $w_1 = 1$. The contraction of the genotype cloud at large recombination rates is seen to persist up to $w_0 = 0.4$ for the chosen parameters, showing that lethality of the deleterious mutations is not a necessary condition for the results presented in the main text.
