# Supplementary material for: Conflicting effects of recombination on the evolvability and robustness in neutrally evolving populations
Source: PLoS Comput Biol. 2022 Nov 21;18(11):e1010710. doi: 10.1371/journal.pcbi.1010710 (PMC9721492; doi:10.1371/journal.pcbi.1010710)
Supplement: S5 Fig — Dependence of evolvability measures on recombination rate obtained with the uniform crossover scheme employed in the main text is compared to results obtained with a one-point crossover scheme. The right column with U = 0.1 is identical to that of Fig 9. In order to implement the one-point crossover in the ism, each novel mutation is assigned a genomic position as a uniform random variable within the range [0, 1), and the position of the crossover is determined by another random number within this range. The non-monotonic behavior persists, but the measures vary more slowly with r. This is likely due to the fact that the single-point crossover produces less diversity compared to the uniform crossover. (PDF) [file pcbi.1010710.s006.pdf]

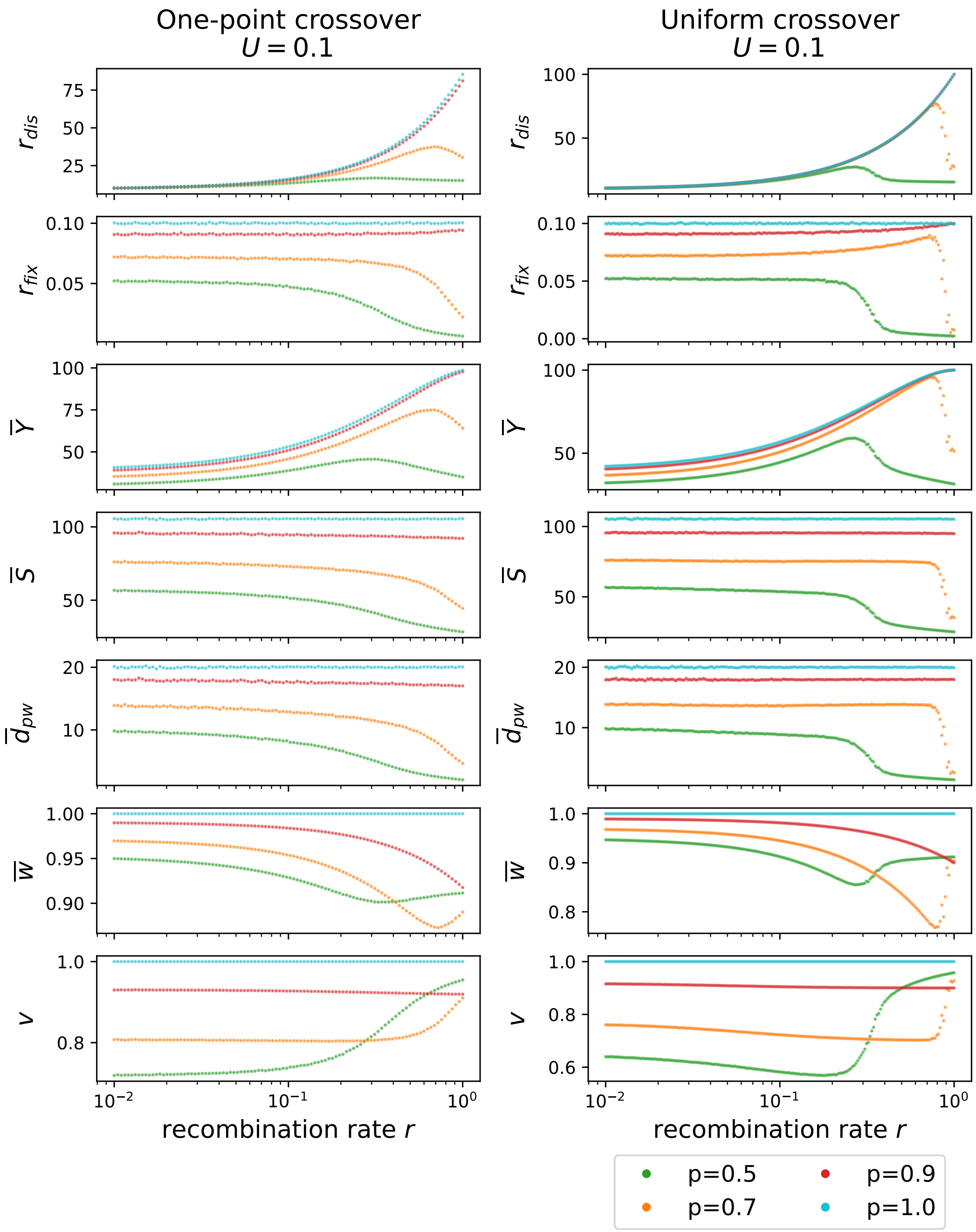

FIG. S5. **One-point crossover vs. uniform crossover.** Dependence of evolvability measures on recombination rate obtained with the uniform crossover scheme employed in the main text is compared to results obtained with a one-point crossover scheme. The right column with $U = 0.1$ is identical to that of Fig. 9. In order to implement the one-point crossover in the *ism*, each novel mutation is assigned a genomic position as a uniform random variable within the range $[0, 1)$, and the position of the crossover is determined by another random number within this range. The non-monotonic behavior persists, but the measures vary more slowly with r. This is likely due to the fact that the single-point crossover produces less diversity compared to the uniform crossover.
