# Supplementary material for: Conflicting effects of recombination on the evolvability and robustness in neutrally evolving populations
Source: PLoS Comput Biol. 2022 Nov 21;18(11):e1010710. doi: 10.1371/journal.pcbi.1010710 (PMC9721492; doi:10.1371/journal.pcbi.1010710)
Supplement: S6 Fig — The right column with U = 0.1 is identical to that in Fig 9 and S5 Fig. Whereas in the main text the recombination rate r describes the recombining fraction of the population, in the left column all individuals recombine and r describes instead the average number of crossovers, which is assumed to be Poisson distributed. Crossovers are implemented as described in the caption of S5 Fig, with the additional feature that multiple crossovers can occur. The results show that a non-monotonic dependence of r can still arise but, similar to S5 Fig, the variation with r occurs more slowly. (PDF) [file pcbi.1010710.s007.pdf]

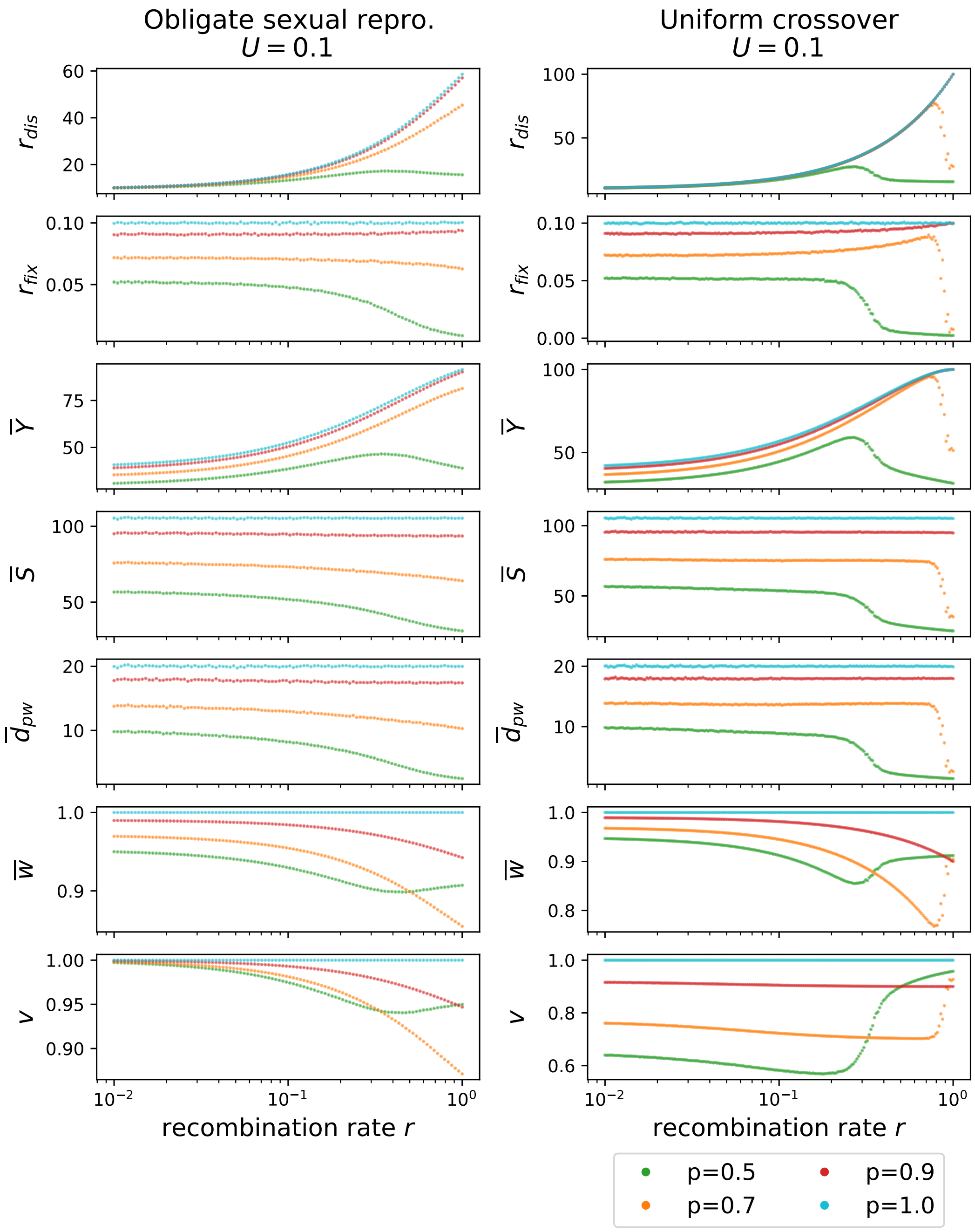

FIG. S6. **Obligate vs. facultative sexual reproduction.** The right column with $U = 0.1$ is identical to that in Figs. 9 and S5. Whereas in the main text the recombination rate r describes the recombining fraction of the population, in the left column all individuals recombine and r describes instead the average number of crossovers, which is assumed to be Poisson distributed. Crossovers are implemented as described in the caption of Fig. S5, with the additional feature that multiple crossovers can occur. The results show that a non-monotonic dependence of r can still arise but, similar to Fig. S5, the variation with r occurs more slowly.
