# Supplementary material for: Conflicting effects of recombination on the evolvability and robustness in neutrally evolving populations
Source: PLoS Comput Biol. 2022 Nov 21;18(11):e1010710. doi: 10.1371/journal.pcbi.1010710 (PMC9721492; doi:10.1371/journal.pcbi.1010710)
Supplement: S7 Fig — Parameters are N = 100, L = 10, p = 0.5. The green line is drawn at μ = 0.01 in both panels. Similar to the results for the ism (Fig 8), the fitness displays an intermediate minimum at the point where the population structure changes. This is best visible at μ = 0.03 (blue line). Compared to the ism the variation in mean fitness and viable recombination fraction is less pronounced. (PDF) [file pcbi.1010710.s008.pdf]

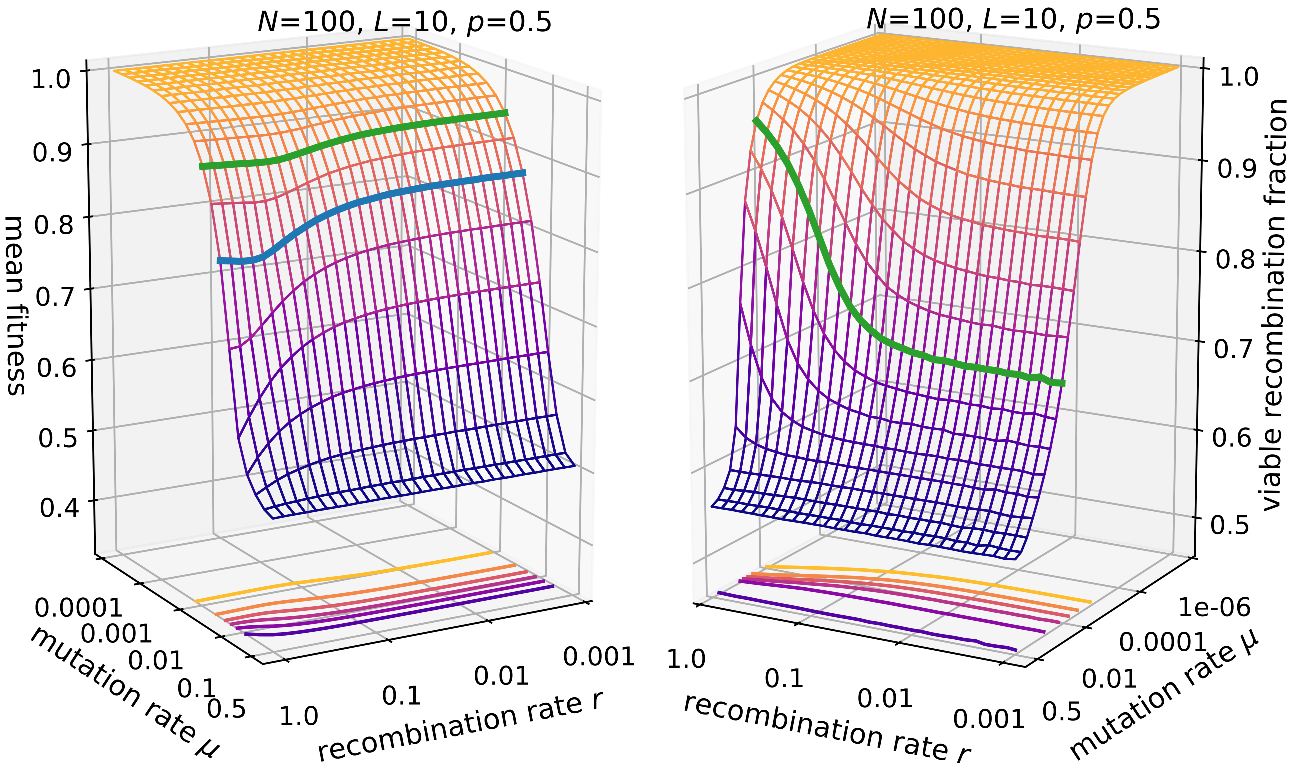

FIG. S7. **Mean fitness and viable recombination fraction in the *fsm*.** Parameters are $N = 100$, $L = 10$, $p = 0.5$. The green line is drawn at $\mu = 0.01$ in both panels. Similar to the results for the *ism* (Fig. 8), the fitness displays an intermediate minimum at the point where the population structure changes. This is best visible at $\mu = 0.03$ (blue line). Compared to the *ism* the variation in mean fitness and viable recombination fraction is less pronounced.
