# Supplementary material for: Conflicting effects of recombination on the evolvability and robustness in neutrally evolving populations
Source: PLoS Comput Biol. 2022 Nov 21;18(11):e1010710. doi: 10.1371/journal.pcbi.1010710 (PMC9721492; doi:10.1371/journal.pcbi.1010710)
Supplement: S8 Fig — Here the time is measured until a randomly chosen escape variant is found by at least one individual in the fsm. Top panel shows the inverse discovery time averaged over all simulations, as well as the inverse time for a subset of simulations where the escape variant has either robustness mescape = 1.0 or mescape = 0.6. These subsets represent scenarios where the robustness of the escape variant is either larger or lower than the average robustness p = 0.8. The results show that more robust genotypes are detected more quickly and less robust genotypes less quickly. The bottom panel shows analogous results where the subsets are distinguished according to the robustness of the initial genotype. In this case the discovery time is not affected. (PDF) [file pcbi.1010710.s009.pdf]

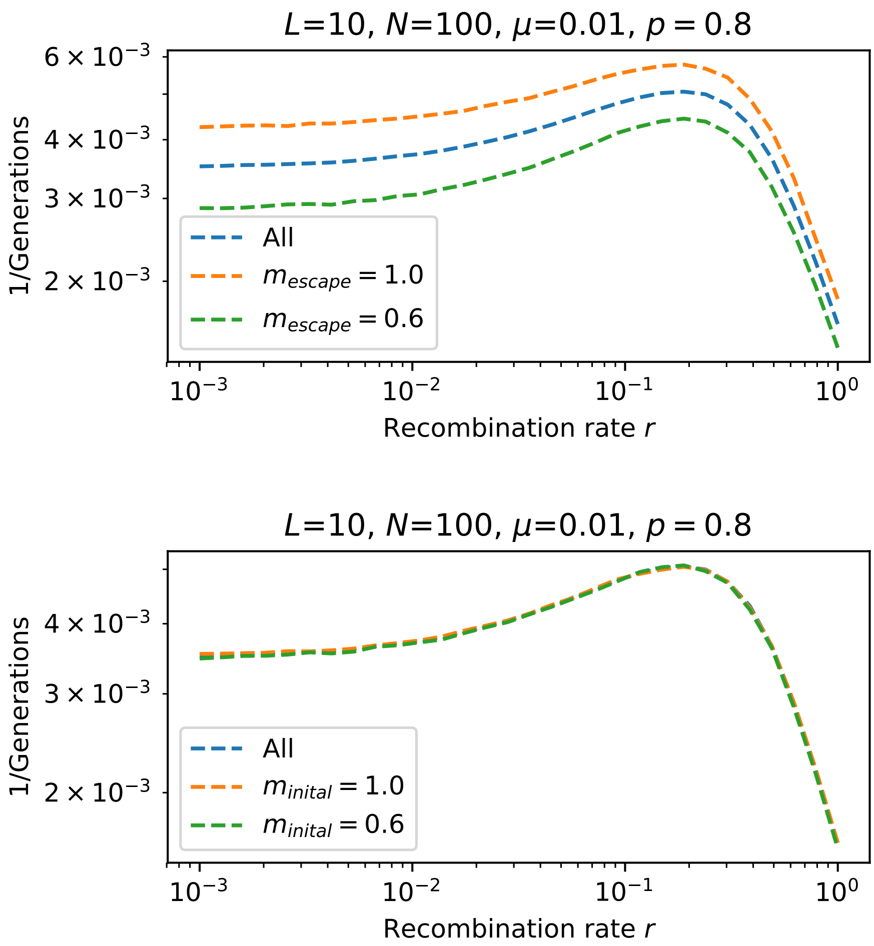

FIG. S8. **Discovery rate of an escape variant.** Here the time is measured until a randomly chosen escape variant is found by at least one individual in the *fsm*. Top panel shows the inverse discovery time averaged over all simulations, as well as the inverse time for a subset of simulations where the escape variant has either robustness $m_{escape} = 1.0$ or $m_{escape} = 0.6$. These subsets represent scenarios where the robustness of the escape variant is either larger or lower than the average robustness $p = 0.8$. The results show that more robust genotypes are detected more quickly and less robust genotypes less quickly. The bottom panel shows analogous results where the subsets are distinguished according to the robustness of the initial genotype. In this case the discovery time is not affected.
