# Supplementary material for: Conflicting effects of recombination on the evolvability and robustness in neutrally evolving populations
Source: PLoS Comput Biol. 2022 Nov 21;18(11):e1010710. doi: 10.1371/journal.pcbi.1010710 (PMC9721492; doi:10.1371/journal.pcbi.1010710)
Supplement: S9 Fig — Parameters are N = 100, L = 10 and p = 1.0 (left panel) vs. p = 0.5 (right panel). The green line is drawn at Lμ = 0.1. (PDF) [file pcbi.1010710.s010.pdf]

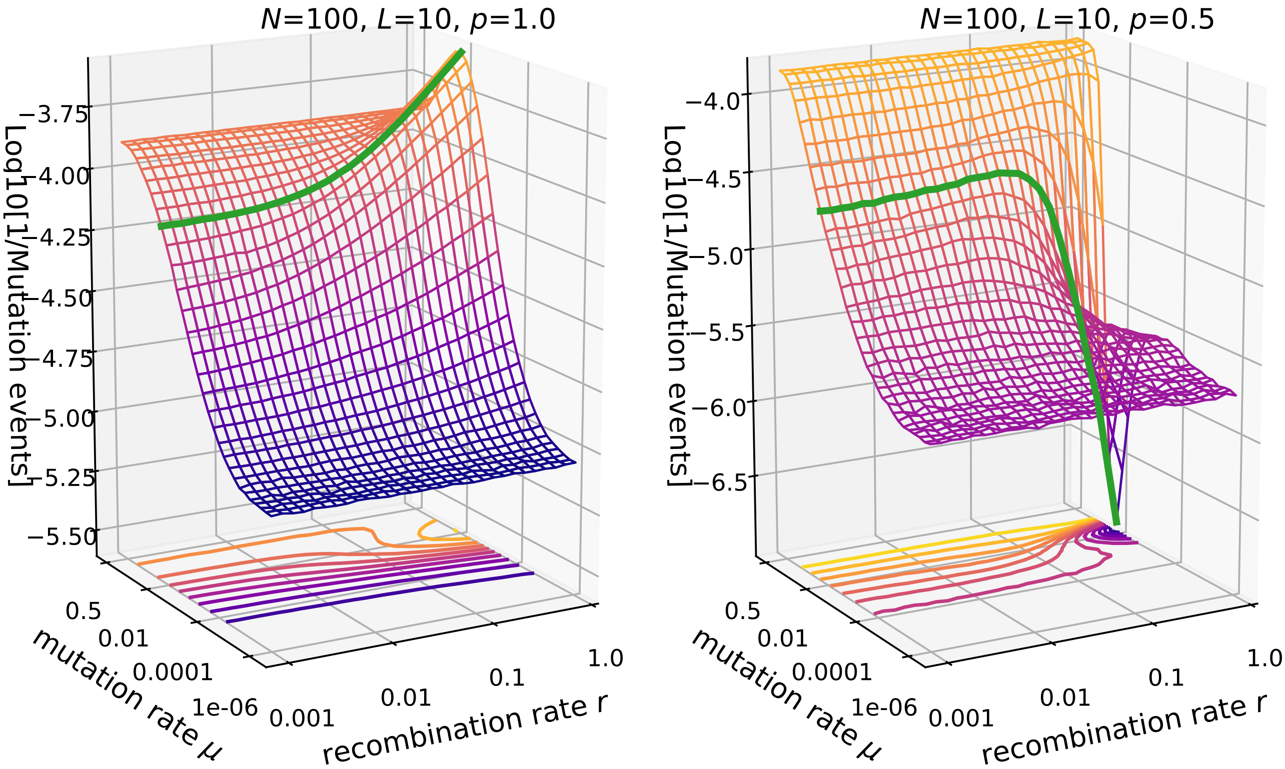

FIG. S9. **Reciprocal of the total number of mutation events until full discovery in the *fsm*.** Parameters are $N = 100$, $L = 10$ and $p = 1.0$ (left panel) vs. $p = 0.5$ (right panel). The green line is drawn at $L\mu = 0.1$.
