# Supplementary material for: Conflicting effects of recombination on the evolvability and robustness in neutrally evolving populations
Source: PLoS Comput Biol. 2022 Nov 21;18(11):e1010710. doi: 10.1371/journal.pcbi.1010710 (PMC9721492; doi:10.1371/journal.pcbi.1010710)
Supplement: S11 Fig — Supplementary information to Fig 13. The population has evolved for 107 generations. Inset histograms show the frequency distribution sorted by rank. The histograms are in semi-log scale for r = 0 and in log-log scale for r − 1. Through the number of ranks, the histograms also display the number of existing distinct genotypes. Note that for both values of p recombination makes the frequency distribution heavy-tailed, but it may either increase or decrease the number of distinct genotypes. Except in the upper right panel (r = 1, p = 1), the node sizes are normalized such that the smallest and largest nodes in each panel have the same size. (PDF) [file pcbi.1010710.s012.pdf]

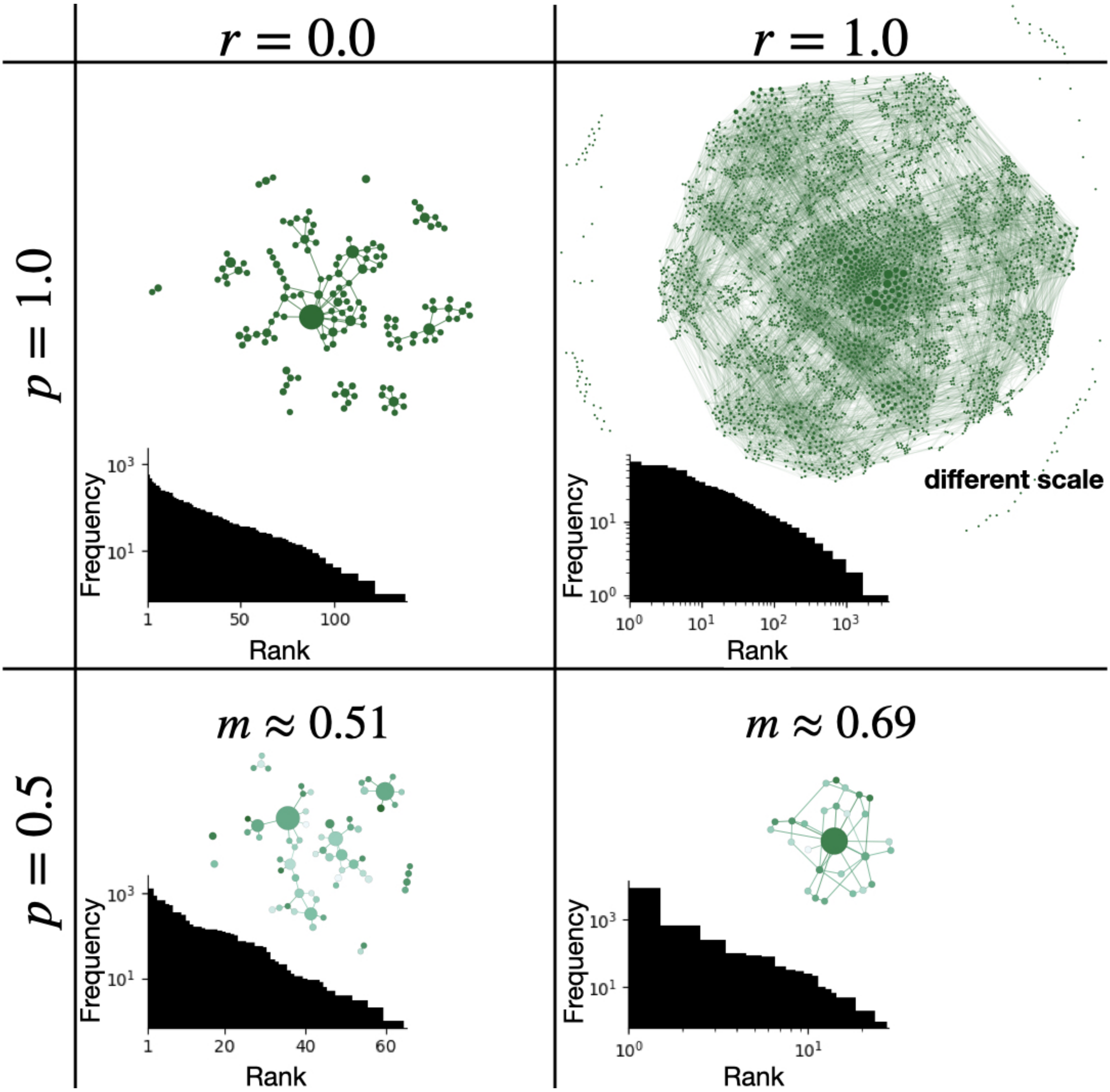

FIG. S11. **Graph representation of genotype clouds in the *fsm* with** $N = 10000$, $L = 14$, $\mu = 0.0001$. Supplementary information to Fig. 13. The population has evolved for 10^7 generations. Inset histograms show the frequency distribution sorted by rank. The histograms are in semi-log scale for $r = 0$ and in log-log scale for $r - 1$. Through the number of ranks, the histograms also display the number of existing distinct genotypes. Note that for both values of p recombination makes the frequency distribution heavy-tailed, but it may either increase or decrease the number of distinct genotypes. Except in the upper right panel ($r = 1$, $p = 1$), the node sizes are normalized such that the smallest and largest nodes in each panel have the same size.
