# Supplementary material for: Conflicting effects of recombination on the evolvability and robustness in neutrally evolving populations
Source: PLoS Comput Biol. 2022 Nov 21;18(11):e1010710. doi: 10.1371/journal.pcbi.1010710 (PMC9721492; doi:10.1371/journal.pcbi.1010710)
Supplement: S12 Fig — Each panel compares obligately recombining (r = 1) and non-recombining (r = 0) populations. Thick lines represent the mean over 5000 landscape realizations and the shaded areas the corresponding standard deviation. (PDF) [file pcbi.1010710.s013.pdf]

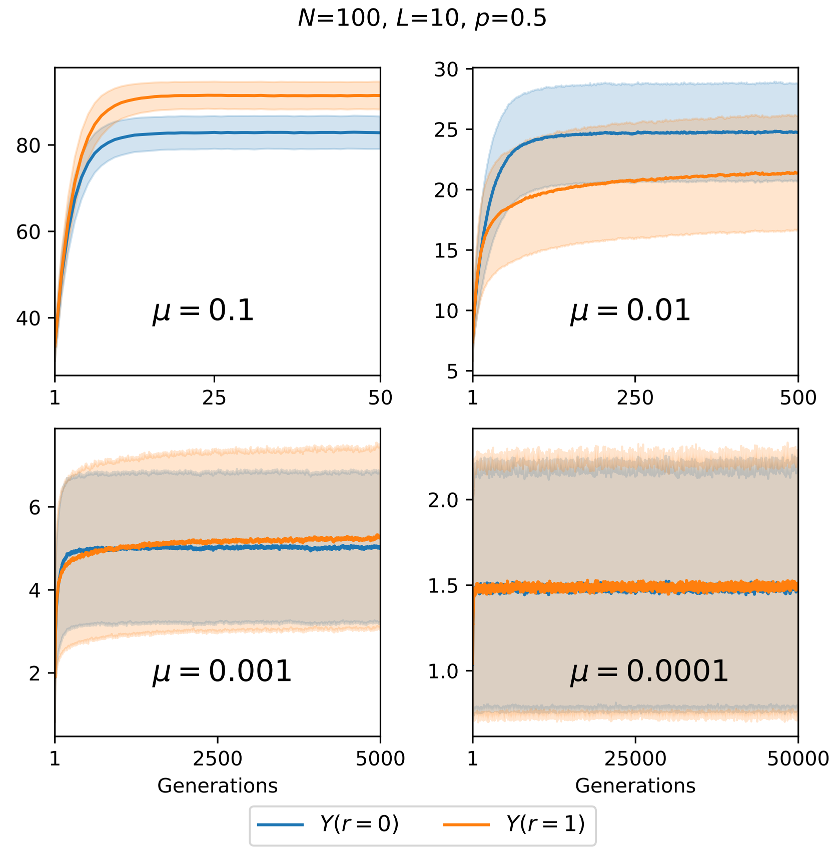

FIG. S12. **Time evolution of the number of distinct genotypes in the *fsm* with $N = 100$, $L = 10$, $p = 0.5$ for different values of the mutation rate μ.** Each panel compares obligately recombining ($r = 1$) and non-recombining ($r = 0$) populations. Thick lines represent the mean over 5000 landscape realizations and the shaded areas the corresponding standard deviation.
