# Supplementary material for: Conflicting effects of recombination on the evolvability and robustness in neutrally evolving populations
Source: PLoS Comput Biol. 2022 Nov 21;18(11):e1010710. doi: 10.1371/journal.pcbi.1010710 (PMC9721492; doi:10.1371/journal.pcbi.1010710)
Supplement: S16 Fig — Parameters are N = 100, L = 10, p = 0.5. The green line at μ = 0.001 (NLμ = 1) shows a non-monotonic variation with recombination rate, which is caused by recombination-dependent genetic drift. (PDF) [file pcbi.1010710.s017.pdf]

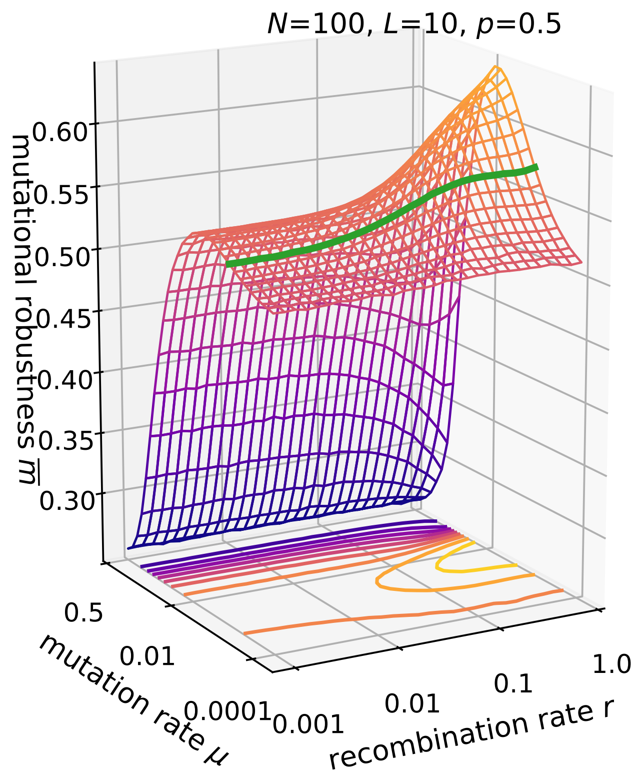

FIG. S16. **Mutational robustness in the *fsm* and simple successive recombination dynamics.** Parameters are $N = 100$, $L = 10$, $p = 0.5$. The green line at $\mu = 0.001$ ($NL\mu = 1$) shows a non-monotonic variation with recombination rate, which is caused by recombination-dependent genetic drift.
