# Supplementary material for: Conflicting effects of recombination on the evolvability and robustness in neutrally evolving populations
Source: PLoS Comput Biol. 2022 Nov 21;18(11):e1010710. doi: 10.1371/journal.pcbi.1010710 (PMC9721492; doi:10.1371/journal.pcbi.1010710)
Supplement: Attachment — Submitted filename: Response_Klug_Krug.pdf [file pcbi.1010710.s018.pdf]

RESPONSE TO REVIEWER COMMENTS

We thank the editors and reviewers for their positive assessment of our manuscript and the constructive comments. A detailed response to Reviewer #1 is provided below. We hope that our revised manuscript is now acceptable for publication in PLoS Computational Biology.

Sincerely,

Alexander Klug and Joachim Krug

Reviewer #1:

However, I have one major concern. Currently, there is no real discussion on how their results map onto real systems. I think it will greatly benefit from some connection to reality. For example, the populations they talk about are facultative sexual (for example, yeast). Could they compare the parameters and dimensionless numbers in their simulations with real life evolutionary parameters of facultatively sexual organisms? Right now, it is not clear whether the recombination rates where the tradeoff is observed are too high for realistic populations. If that is the case, then the study becomes less interesting as it is exploring parameter regimes that are not realistic.

In short, I would like the authors to address how their work relates to real organisms, at least in the discussion section.

Response: We thank the reviewer for this important remark. In fact, the effects we describe do not depend on the recombination rate in absolute terms, but rather on the ratio r/U. We therefore expect our results to be principally applicable also to facultative sexual species with rare sex. We have added a paragraph to the Discussion section where we explain this point and refer to some relevant literature (Refs. 71-74).

Additional changes

- A citation to a review article on facultative sexuals (Ref. 56) has been added on page 5.
- Equation (20) has been moved from the SI Appendix to the main text.
- Supplementary Figure S17 has been moved to SI Appendix.
- The supplementary material has been reorganized to conform with the rules of PLoS CB.
